# Contributions to the Journal of Comparative Medicine

**Published:** 1881-04

**Authors:** 


CONTRIBUTIONS TO THE JOURNAL OF COMPARATIVE MEDICINE. We shall be glad to receive communications from veterinarians on subjects relating to the diseases of animals. There is a disinclination among many veterinarians to write, even if it is only to report cases. This apathy is a thing which is greatly to be deplored. Nothing assists so much in forming habits of careful observation, and in gaining skill in diagnosis, as the recording of cases. We trust that our subscribers will take the trouble to pay more attention to this subject. Articles need not be long or elaborate. The simple history of cases often furnishes the most instructive and suggestive kind of medical writing.

We have been pleased to find that with each issue of the JOURNAL a larger number of communications has been sent us. We trust that the increase will continue.

